# Two unlike cousins: *Candida albicans* and *C. glabrata* infection strategies

**DOI:** 10.1111/cmi.12091

**Published:** 2013-01-14

**Authors:** Sascha Brunke, Bernhard Hube

**Affiliations:** 1 Department of Microbial Pathogenicity Mechanisms, Leibniz Institute for Natural Product Research and Infection Biology – Hans Knoell Institute Jena (HKI)Beutenbergstrasse 11a, 07745, Jena, Germany; 2 Integrated Research and Treatment Center, Sepsis und Sepsisfolgen, Center for Sepsis Control and Care (CSCC), Universitätsklinikum JenaJena, Germany; 3 Friedrich Schiller UniversityJena, Germany

## Abstract

*Candida albicans* and *C. glabrata* are the two most common pathogenic yeasts of humans, yet they are phylogenetically, genetically and phenotypically very different. In this review, we compare and contrast the strategies of *C. albicans* and *C. glabrata* to attach to and invade into the host, obtain nutrients and evade the host immune response. Although their strategies share some basic concepts, they differ greatly in their outcome. While *C. albicans* follows an aggressive strategy to subvert the host response and to obtain nutrients for its survival, *C. glabrata* seems to have evolved a strategy which is based on stealth, evasion and persistence, without causing severe damage in murine models. However, both fungi are successful as commensals and as pathogens of humans. Understanding these strategies will help in finding novel ways to fight *Candida*, and fungal infections in general.

## Introduction

Fungi infect billions of people every year, but still remain largely under-appreciated as pathogens of humans (Brown *et al*., 2012). In fact, some fungal diseases have an extremely high mortality rate and fungi kill at least as many people as tuberculosis or malaria (Brown *et al*., 2012). Over the last decades, they have become a major problem especially in the clinical setting (Perlroth *et al*., 2007). *Candida albicans* and *C. glabrata*, for example, are ubiquitous commensals of humans, and can be found especially in the oral cavity and the gastrointestinal tract of most healthy humans (Cole *et al*., 1996; Fidel *et al*., 1999). On the other hand, they are also the most important pathogenic yeasts. The majority of the population is asymptomatically colonized by either of the two species, or even both (Li *et al*., 2007b). However, under certain predisposing factors, such as treatment with antibiotics, diabetes, cancer, extreme age, immunosuppression, intravenous catheters or long-term hospitalization, these fungi can lead to superficial or even life-threatening systemic infections, with high morbidity and mortality (Perlroth *et al*., 2007). Among the *Candida* species, *C. albicans* and *C. glabrata* rank first and second in isolation frequency, respectively, and, together, are responsible for approximately 65%–75% of all systemic candidiasis, followed by *C. parapsilosis* and *C. tropicalis* (Perlroth *et al*., 2007).

While *C. albicans, C. parapsilosis* and *C. tropicalis* are relatively closely related members of the so-called CUG clade (sharing a unique codon exchange from leucine to serine), *C. glabrata* is, as a *Candida* species, in fact a ‘misnomer’: this yeast is actually much more closely related to the baker's yeast *Saccharomyces cerevisiae* than to *C. albicans* (Dujon *et al*., 2004), and this is reflected by a number of differences. For example, unlike *C. albicans*, the progenitor of *C. glabrata* and *S. cerevisiae* experienced a whole-genome duplication event (Dujon *et al*., 2004). Additionally, *C. albicans* is a diploid, polymorphic fungus, switching readily from yeast to hyphal (and pseudohyphal) growth and back. In contrast, *C. glabrata* is strictly haploid and normally grows only in the yeast form (Kaur *et al*., 2005). The morphological flexibility of *C. albicans* seems to play fundamental roles in several aspects of infection (Sudbery, 2011). In contrast, the pathogenicity of *C. glabrata* seems to be independent of morphology. Yet, both fungi are closely associated with humans, and similarly successful as commensals and as pathogens. But how similar are their commensal and pathogenic strategies?

The phylogenetic tree, in which *C. albicans* (and the CUG clade) and *C. glabrata* are separated by several non-pathogenic yeasts, strongly implies that the ability to infect humans has evolved independently in the two species. Therefore, a closer look at the similarities and differences in the persistence and infection strategies of *C. albicans* and *C. glabrata* may help to understand general principles of fungal infections.

## Adhesion

For any successful commensal and pathogen, adhesion to host cells is essential. Many bacteria possess elaborate systems to detect the presence of the host by environmental cues, in order to express specific adhesins (Kline *et al*., 2009). These factors in turn allow the microorganisms to attach firmly to a host cell, and help to prevent them from being washed away. Adhesins are also critical for the formation of biofilms: Both *C. albicans* and *C. glabrata* can form biofilms on abiotic substrates, especially medical devices such as catheters (Iraqui *et al*., 2005; Nobile and Mitchell, 2006).

*Candida albicans* and *C. glabrata* have large protein families of adhesins at their disposal: for *C. albicans*, the Als proteins with their Agglutinin-Like Sequences, are crucial adhesins (Hoyer *et al*., 2008). Especially the Als3 protein seems to play a vital role in adhesion, since its deletion strongly reduces adhesion to epithelial cells (Hoyer *et al*., 2008; Wächtler *et al*., 2012). In addition, this protein is also important for iron acquisition from ferritin (Almeida *et al*., 2008), formation of mixed-species biofilms (Silverman *et al*., 2010) and induction of endocytosis by host cells (Phan *et al*., 2007; Wächtler *et al*., 2012 and see also next section). As Als3 is hypha-associated (Argimon *et al*., 2007), it is expressed as the fungus forms filaments, for example upon physical contact with host cells, at body temperature and at ambient neutral pH. Expression of another important adhesin, Hwp1, is likewise hypha-associated (Staab *et al*., 1999), strengthening the concept of induced, hypha-associated adhesion, as opposed to the ‘*ad hoc*’ non-induced adhesion of attaching yeast cells (Wilson and Hube, 2010; Wächtler *et al*., 2011).

The main adhesins of *C. glabrata*, the Epa proteins, are related to the Flo proteins of *S. cerevisiae*, which are responsible for flocculation during the brewing process. In *C. glabrata*, this family of approximately 17–23 genes (depending on the isolate) allows attachment to epithelial cells (Cormack *et al*., 1999; Castano *et al*., 2005) and macrophages (Kuhn and Vyas, 2012). The predominant adhesin during *in vitro* interaction with epithelial cells is Epa1 (Cormack *et al*., 1999), whereas other Epa and non-Epa adhesins can mediate attachment to other cell types (de Groot *et al*., 2008; Desai *et al*., 2011; Kraneveld *et al*., 2011). Additionally, it has been suggested that the high expression heterogeneity of Epa1, and likely other adhesins, leads to distinct subsets of Epa-expressing cells in any population, each with different adhesion properties (Halliwell *et al*., 2012).

A unique mechanism for sensing the host environment has been described for the genes encoding *C. glabrata*'s Epa proteins: their expression is regulated by Sir-complex-mediated transcriptional silencing (De Las Penas *et al*., 2003; Castano *et al*., 2005). This system requires NAD^+^ as a cofactor, and since *C. glabrata* is auxotrophic for the NAD^+^ precursor nicotinic acid (NA), the silencing activity is indirectly influenced by external NA concentrations. Because urine is low in nicotinic acid, *EPA* genes are derepressed in the urinary tract, promoting adhesion of *C. glabrata* in this niche (Domergue *et al*., 2005). Interestingly, no systematic studies have so far investigated whether the physiological body temperature has similar effects on the transcriptional programme, as is clearly the case in *C. albicans*.

In summary, both fungi have independently evolved specific adhesins which rely on different cues to detect the presence of the host. While the details differ, the basic principle is the same – upon detection of a ‘host’ environment, adhesins are expressed to attach to host cells.

## Invasion

After attachment, the next step in *Candida* pathogenesis is invasion, normally into epithelial cell layers. For *C. albicans*, invasion can occur via two mechanisms: induced endocytosis by host cells, and active penetration by *C. albicans* hyphae (Wächtler *et al*., 2011b^2012^). Als3 is one of the invasins which trigger endocytosis by inducing host cytoskeletal rearrangements (Phan *et al*., 2007). This process does not require viable fungi and does not cause damage *per se*, but seems to be important at the early stages of invasion (Wächtler *et al*., 2011; 2012; Zhu *et al*., 2012). Yet, the dominant route of invasion is active penetration of host cells. Hyphae penetrate tissue by a combination of physical forces exerted by the extending filaments, the secretion of hydrolytic enzymes and as yet unknown damaging factors, which finally leads to disruption of host cell membranes (Wächtler *et al*., 2012). However, hyphae formation comes at a price: epithelial cells react to the hyphal surface, and to the inflicted damage, by secreting pro-inflammatory cytokines, demarcating the transition to a pathogenic lifestyle (Schaller *et al*., 2002; Moyes *et al*., 2010b^2012^). These events in turn attract macrophages and neutrophils (Schaller *et al*., 2004), which can fight and kill invading *C. albicans*.

Hypha-mediated penetration is widely assumed to play an important role in gaining access to deeper tissue and the bloodstream. How can *C. glabrata* enter the circulatory system without hyphae? A possible route to reach the bloodstream is the accidental or iatrogenic breach of natural barriers via trauma, catheters, surgery or parenteral nutrition (Perlroth *et al*., 2007). However, even in the absence of such breaches, *C. glabrata* yeasts invade into deeper tissues and readily disseminate in a chicken embryo model of fungal infections (Jacobsen *et al*., 2011). The mode of this invasion is unclear, although *S. cerevisiae* cells are known to form agar-invasive pseudohyphal under *in vitro* starvation conditions (Gimeno *et al*., 1992) and one report even described pseudohyphae of *C. glabrata in vitro* (Csank and Haynes, 2000). However, *in vivo*, it may rely on endocytosis, in lieu of active penetration, with close to no host cell damage (Li *et al*., 2007a). Most likely because of low host damage, the cytokine profile of epithelia infected with *C. glabrata* differs dramatically from that of *C. albicans*-infected cells: *C. glabrata* induces more GM-CSF than *C. albicans*, but nearly none of the other inflammatory cytokines like IL-1α or Il-8 (Schaller *et al*., 2002; Li *et al*., 2007a). This is in good agreement with the cytokine pattern observed during *in vivo* murine infections (Jacobsen *et al*., 2010). Consequently, strong neutrophil infiltration is characteristic for *C. albicans* infections, while *C. glabrata* either does not stimulate or is able to suppress neutrophil attraction and is rather associated with mononuclear cells (Westwater *et al*., 2007; Jacobsen *et al*., 2010). Overall, the presence of hyphae and host cell damage in *C. albicans* infections leads to a stronger pro-inflammatory cytokine response than in *C. glabrata* infections.

## Interaction with immune cells

Macrophages are part of the first line of defence by the innate immune system. These phagocytes recognize invading fungi in the tissue via a subset of their PRRs (pattern recognition receptors) which specifically bind to fungal PAMPs (pathogen associated molecular pattern), such as β-glucan (Taylor *et al*., 2007), mannan or chitin (van der Meer *et al*., 2010; Mora-Montes *et al*., 2011). Interestingly, *C. glabrata* is recognized and ingested by macrophages at a much higher rate than *C. albicans*, and *C. albicans* yeasts more than hyphae (Keppler-Ross *et al*., 2010). These preferences seem to be mannan dependent, but independent of glucan or chitin (Keppler-Ross *et al*., 2010). Without intervention by the phagocytosed microbe, the phagosome normally matures via a series of fusion and fission events, and the pathogens are killed in the matured phagolysosome. Furthermore, by releasing cytokines upon recognition of pathogens, macrophages help to orchestrate the immune responses.

However, when *C. glabrata* is internalized by macrophages, it modifies the normal phagosome maturation process. It remains in a non-acidified organelle which lacks typical lysosomal markers such as cathepsin D (Seider *et al*., 2011). Similar to *Histoplasma capsulatum* (Woods, 2003; Seider *et al*., 2010) and bacteria like *Mycobacterium* spp. (de Chastellier, 2009), *C. glabrata* not only survives, but replicates inside the phagosome until the phagocyte finally bursts (Kaur *et al*., 2007; Roetzer *et al*., 2010; Seider *et al*., 2011). Other survival strategies include expression of a highly active catalase (Cuellar-Cruz *et al*., 2008) and pigment production, which may counteract the oxidative burst of phagocytes (Brunke *et al*., 2010). In addition, the cytokine response of macrophages to *C. glabrata in vitro* is much lower than during interaction with *C. albicans* (Seider *et al*., 2011). Consistently, murine and chicken embryo infections with *C. glabrata* lead only to a transient pro-inflammatory cytokine response, and only a minor influx of immune effector cells (Jacobsen *et al*., 2010; 2011). During *in vitro* experiments, *C. albicans* also appears to delay phagosome maturation and to induce recycling of late maturation markers like LAMP-1 (Fernández-Arenas *et al*., 2009). However, soon after these initial events, *C. albicans* starts to form hyphae which disrupt the macrophage membranes, effectively killing the phagocyte and allowing the fungus to rapidly escape (Lo *et al*., 1997; McKenzie *et al*., 2010). The macrophage phagosome is a nutrient-poor and harmful environment. Escape from phagocytes may therefore be a strategy of *C. albicans* to quickly escape this detrimental environment (Hummert *et al*., 2010), while *C. glabrata* depends on fungal autophagy to survive inside macrophages (Roetzer *et al*., 2010). Interestingly, even autophagy-deficient mutants of *C. albicans* can still escape the phagocytes and subsequently gain access to the external nutrients (Palmer *et al*., 2007). The *in vitro* observations of a quick escape from macrophages by *C. albicans* have been challenged recently by experiments in live zebrafish. In this model, *C. albicans* remained viable after phagocytosis, but hyphae formation was not observed (Brothers *et al*., 2011). How far this reflects the situation in the mammalian host, however, is largely unknown, especially since mammals differ from the ectothermic zebrafish model by their high body temperature – an important hypha-inducing factor. Additionally, a recent study even reported a rare non-lytic escape of *C. albicans* yeasts cells from macrophages (Bain *et al*., 2012), in a manner reminiscent of the process described for *Cryptococcus neoformans* (Alvarez and Casadevall, 2006). Thus, the precise *in vivo* escape strategies of *C. albicans* from phagocytes require further research.

## Nutrient acquisition within the host

During infection, a molecular tug-of-war takes place between the host, which tries to restrict access to essential nutrients, and the pathogen, which needs these nutrients to survive and multiply. Acquisition of nutrients by the fungus is therefore central to establishing and maintaining infection. Interestingly, due to its frequent gene losses (Dujon *et al*., 2004), *C. glabrata* lacks many of the metabolic pathways known in other yeasts, including *S. cerevisiae* and *C. albicans*. It cannot catabolize galactose (due to its loss of genes *GAL1*, *7*, *10*) and allantoin (*DAL1–4, 7*), and is auxotrophic for pyridoxine (*SNO1, 2, 3*), nicotinic acid (*BNA1–6*) and thiamine (Dujon *et al*., 2004; Wong and Wolfe, 2005). These restrictions must be overcome in the specific host niches conquered by *C. glabrata*. In contrast, *C. albicans* does not have any known auxotrophies, can metabolize a broad range of sugars and can use all amino acids as sole nitrogen sources (Odds, 1988; Kaur *et al*., 2005; and own observations). In addition, *C. albicans* possesses several families of secreted hydrolases and transmembrane transporters with central roles in virulence (Butler *et al*., 2009). Secreted aspartic proteases (Saps), for example, have the potential to destroy host tissue and liberate oligopeptides and amino acids, which are taken up by the fungus via oligopeptide and amino acid transportes (Naglik *et al*., 2004). Contrarily, *C. glabrata* exhibits low extracellular protease activity *in vitro* (Kaur *et al*., 2005).

Micronutrients are another prerequisite for a successful infection. Especially metals like iron and zinc are subject to a process called ‘nutritional immunity’, where the host actively sequesters these elements from invading microorganisms (Hood and Skaar, 2012). Iron levels in human serum are held as low as 10^−24^ M, severely restricting its availability to pathogens. To counteract this limitation, *C. albicans* has a plethora of iron acquisition systems at its disposal (Almeida *et al*., 2009): It can utilize siderophores from other microorganisms without producing its own (via Sit1/Arn1, Heymann *et al*., 2002), and bind host transferrin (via an unknown receptor, Knight *et al*., 2005) and ferritin (via Als3, Almeida *et al*., 2008). In addition, *C. albicans* can express haemolysins that disrupt red blood cells (Watanabe *et al*., 1999) and then bind and utilize haemoglobin via the Rbt5/Hmx1 system (Pendrak *et al*., 2004; Weissman and Kornitzer, 2004). Free iron, if available, is taken up via the reductive pathway with its large gene families of reductases, oxidases and iron permeases (Almeida *et al*., 2009). Together, these systems enable *C. albicans* to effectively use nearly all natural iron sources both of the host during infection and of surrounding microbes during commensal growth.

*Candida glabrata*, on the other hand, has no known receptors for haem (Nevitt and Thiele, 2011); however, haemolysin expression has been reported (Luo *et al*., 2004). In addition, although the reductive pathway is present, *C. glabrata* is not known to use host ferritin or transferrin as iron sources. Interestingly, like *C. albicans*, it can bind hydroxamate-type xenosiderophores of fungal origin, like ferrichrome, ferrirubin or coprogen, but unlike *C. albicans* not bacterial siderophores (Nevitt and Thiele, 2011). This binding significantly increases fitness and survival inside macrophages after subsequent phagocytosis (Nevitt and Thiele, 2011). Therefore, *C. glabrata*'s *in vivo* choice of iron sources would appear to be limited in comparison with *C. albicans*.

Zinc, a central cofactor for many proteins, is also actively limited by the host during infections (Corbin *et al*., 2008), but it can be scavenged by *C. albicans* via a recently discovered ‘zincophore’ system using Pra1 (Citiulo *et al*., 2012). This protein is secreted, binds zinc and delivers it back to the pathogen in a manner reminiscent of the iron-carrying siderophores. *C. glabrata* is missing both Pra1 and its proposed binding partner, the high-affinity zinc transporter Zrt1 (Citiulo *et al*., 2012). Possibly, *C. glabrata* acquires Zn via its two homologues of the low-affinity *S. cerevisiae* zinc transporter Zrt2, but this still requires experimental confirmation. In addition, other micronutrients like manganese (Kehl-Fie and Skaar, 2010) or copper (Hodgkinson and Petris, 2012) may play important roles in host–pathogen interactions, and their uptake systems in *Candida* species are still largely unknown.

It seems, therefore, that the high metabolic flexibility of *C. albicans* may be part of its infection strategy, enabling this fungus to survive and grow in the many different and changing host niches it encounters. *C. glabrata*, on the other hand, appears more specialized in its metabolic requirement, possibly requiring a more stable environment, where these needs are met.

## Conclusion

**Fig. 1:**
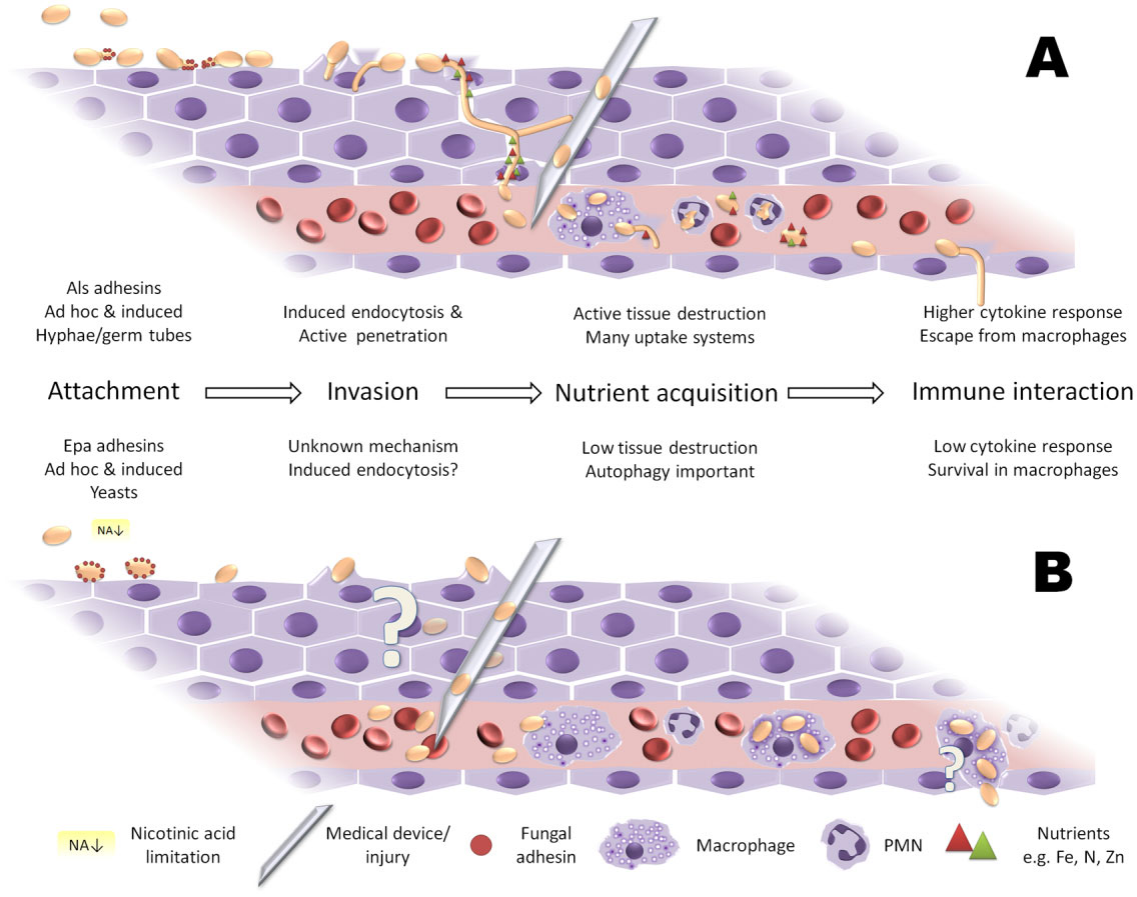
Schematic overview of the two different infection strategies of *C. albicans* and *C. glabrata*. A. *C. albicans* forms hyphae and aggressively destroys tissue, eliciting a strong immune response. B. Many aspects of *C. glabrata* pathogenicity are still unknown, like the precise mechanism of invasion. Active host tissue damage is low, as is the immune response. For a detailed description of the individual steps, see the related sections in the text.

The problems and obstacles faced by both *C. albicans* and *C. glabrata* during infections are essentially the same, but the solutions employed differ in many ways (Fig. 1): overall, *C. glabrata* seems to follow a strategy of stealth and concealment in infection. It does not cause extensive epithelial damage, probably due to its lack of an invasive growth form. It does not elicit a strong immune response in murine models or in *in vitro* reconstituted human epithelia, and it can reside within macrophages without immediately destroying them. When it comes to nutrient supply in the host, *C. glabrata* relies on autophagy and some so far uncharacterized nutrient uptake mechanisms, but it would not appear to elicit rapid tissue damage to release nutrients from host cells. Genomically, *C. glabrata* displays some of the hallmarks of a specialized commensal or pathogen of humans. The loss of many metabolic pathways is indicative of a more stable environment than the one faced by *C. albicans*. Although it is unclear how far these (mostly) *in vitro* observations translate into infections of the human host, *C. glabrata* can persist within the internal organs of mice for a surprisingly long time without any clinical symptoms. A possible scenario is that *C. glabrata* attracts macrophages, which it subverts to use as a ‘trojan horse’, to hide from immune surveillance and to spread to the organs, analogous maybe to the strategy employed by *C. neoformans* (Charlier *et al*., 2009).

*Candida albicans*, on the other hand, follows a strategy which can be described as ‘shock and awe’ when it has changed from commensal to pathogen. It actively invades epithelia when the circumstances permit and elicits stronger immune responses, which it seems to counteract in many cases. Macro- and micronutrients from damaged host tissue are taken up by a broad range of acquisition systems, and defending macrophages may be killed by formation of hyphae. Tissue damage by the fungus and the activated immune system can lead to severe disease, and death. In some cases, *C. glabrata* may benefit from this strategy of *C. albicans*. In oral candidiosis, for example, co-infections by both fungi are common (Redding *et al*., 2002; Coco *et al*., 2008), and *C. glabrata* may exploit the tissue destruction caused by *C. albicans* to gain nutrients, and possibly even to access the bloodstream. Moreover, this concept of *C. albicans* paving the way for *C. glabrata* may have serious clinical consequences: once *C. glabrata* reaches internal organs, its inherently high resistance to many commonly used antifungals makes treatment more problematic.

These strategies may be over-simplified for the purpose of this review, and many nuances were necessarily left out. However, much of the evidence shows that these two fungi use very different pathways to obtain the same goal – to survive and proliferate during infection of the human host. Understanding these strategies will hopefully help in finding novel ways to fight *Candida*, and fungal infections in general.
